# Biosynthesis of poly(3-hydroxybutyrateco-3-hydroxy-4-methylvalerate) by Strain Azotobacter chroococcum 7B

**Published:** 2016

**Authors:** A.P. Bonartsev, G. A. Bonartseva, V. L. Myshkina, V. V. Voinova, T. K. Mahina, I. I. Zharkova, S. G. Yakovlev, A. L. Zernov, E. V. Ivanova, E. A. Akoulina, E. S. Kuznetsova, V. A. Zhuikov, S. G. Alekseeva, V. V. Podgorskii, I. V. Bessonov, M. N. Kopitsyna, A. S. Morozov, E. Y. Milanovskiy, Z. N. Tyugay, G. S. Bykova, M. P. Kirpichnikov, K. V. Shaitan

**Affiliations:** Faculty of Biology, M.V.Lomonosov Moscow State University, Leninskie gory, 1-12, Moscow, 119234 , Russia; A.N. Bach Institute of Biochemistry, Research Center of Biotechnology of the Russian Academy of Sciences, 33, bld. 2 Leninsky Ave., Moscow, 119071, Russia; JSC «Institute of Plastics», Petrovskiy proezd, 35, Moscow, 111024, Russia; Federal scientific-clinical center of physics-chemical medicine, Federal medical-biological agency, Malaya Pirogovskaya str., 1a, Moscow, 119435, Russia; Bauman Moscow State Technical University, 5, 2-nd Baumanskaya, Moscow, 105005, Russia; Faculty of Soil Science, M.V.Lomonosov Moscow State University, Leninskie gory, 1-12, Moscow, 119234, Russia

**Keywords:** Azotobacter chroococcum 7B, poly(3-hydroxybutyrate), poly(3-hydroxybutyrate-co-3-hydroxy-4-methylvalerate), biosynthesis, crystallinity, biocompatibility, bone marrow stromal cells

## Abstract

Production of novel polyhydroxyalkanoates (PHAs), biodegradable polymers for biomedical applications, and biomaterials based on them is a promising trend in modern bioengineering. We studied the ability of an effective strain-producer *Azotobacter chroococcum *7B to synthesize not only poly(3-hydroxybutyrate) homopolymer (PHB) and its main copolymer poly(3-hydroxybutyrate-co-3-hydroxyvalerate) (PHBV), but also a novel copolymer, poly(3-hydroxybutyrate-co-3-hydroxy-4-methylvalerate) (PHB4MV). For the biosynthesis of PHB copolymers, we used carboxylic acids as additional carbon sources and monomer precursors in the chain of synthesized copolymers. The main parameters of these polymers’ biosynthesis were determined: strain-producer biomass yield, polymer yield, molecular weight and monomer composition of the synthesized polymers, as well as the morphology of *A. chroococcum *7B bacterial cells. The physico-chemical properties of the polymers were studied using nuclear magnetic resonance spectroscopy (NMR), differential scanning calorimetry (DSC), contact angle test, and other methods. *In vitro *biocompatibility of the obtained polymers was investigated using stromal cells isolated from the bone marrow of rats with the XTT cell viability test. The synthesis of the novel copolymer PHB4MV and its chemical composition were demonstrated by NMR spectroscopy: the addition of 4-methylvaleric acid to the culture medium resulted in incorporation of 3-hydroxy-4-methylvalerate (3H4MV) monomers into the PHB polymer chain (0.6 mol%). Despite the low molar content of 3H4MV in the obtained copolymer, its physico-chemical properties were significantly different from those of the PHB homopolymer: it has lower crystallinity and a higher contact angle, i.e. the physico-chemical properties of the PHB4MV copolymer containing only 0.6 mol% of 3H4MV corresponded to a PHBV copolymer with a molar content ranging from 2.5% to 7.8%. *In vitro *biocompatibility of the obtained PHB4MV copolymer, measured in the XTT test, was not statistically different from the cell growth of PHB and PHBV polymers, which make its use possible in biomedical research and development.

## INTRODUCTION

Intensive development of such biomedical fields as regenerative medicine, bioengineering (including tissue engineering), biopharmaceuticals, and nanobiotechnology has increased demand for the development of new biomaterials, especially biocompatible and biodegradable polymers. A variety of natural and synthetic polymers are used as materials for the manufacture of medical devices and formulations, including polyhydroxyalkanoates (PHAs), polyanhydrides, polyalkylcyanoacrylates, polyphosphazenes, polyphosphoesters, polyorthoesters, some polysaccharides (chitosan, hyaluronic acid, agarose, dextran, alginates, chondroitin sulfate), and proteins (collagen, fibrin, silk fibroin, spidroin, gelatin) [1-5]. These polymers are used in medical implants in reconstructive surgery [4, 5], tissue engineering [3, 6, 7], for creating new dosage forms in biopharmaceutics [8, 9], new dental materials, and they have other applications [1, 2].

Despite the wide range of polymers used in medicine, the vast majority of them are produced by chemical synthesis or isolated from natural raw materials (algae, higher plants, mushrooms, crustaceans, tissues of domestic animals). Unfortunately, the methods used in the chemical synthesis and isolation of polymers from natural raw materials cannot yield the full range of properties required for biomedical polymers. The obtained polymers require deep, and very expensive, purification, must fulfill very narrow requirements for chemical structure and properties, as well as be biologically safe, etc. Additionally, synthetic polymers and the products of their biodegradation may be toxic, while natural polymers may display pronounced immunogenicity or be contaminated with viruses or prion proteins [10, 11].

Biodegradable poly(3-hydroxyalkanoates), poly(3- hydroxybutyrate) (PHB) and its copolymers (according to the Russian chemical nomenclature of macromolecular compounds and IUPAC [13]), attract particular attention among developed and used biomedical polymers. In contrast to natural polymers (chitosan, alginate, dextran, collagen, etc.) and chemically synthesized polymers, PHAs are produced by biotechnological methods that allow one to achieve a high degree of purity, and to control and specify the physico-chemical properties of the biopolymers within narrow limits during their biosynthesis. PHAs have a set of unique properties: high mechanical strength and thermal plasticity that allows easy processing and obtainment of a wide range of products, ability to form composites with synthetic polymers, inorganic materials, and medicinal products, complete biodegradability to non-toxic products, biocompatibility (including hemocompatibility) with human and animal tissues and organs, and environmental safety. Therefore, PHAs are considered promising for use in medicine [13-16].

PHAs also have a unique nanostructure. As partially crystalline compounds, PHAs can form various supramolecular structures, such as lamellae and spherulites. Such a partially crystalline structure and morphology largely defines the biological properties of PHAs, such as the kinetics of its biodegradation [17, 18].

However, PHAs and other polymeric materials, such as PHB homopolymer, can have certain disadvantages, as well: high hydrophobicity and crystallinity, longterm biodegradation and low plasticity, which in some cases severely limits their use as bioengineered materials in medicine, for example for the manufacture of vessel grafts [19, 20]. Therefore, the development of novel biotechnological methods for obtaining new PHB copolymers for biomedical applications with an optimum combination of the physico-chemical and biological properties of the biomaterials produced from them is considered the most promising trend in modern bioengineering [1, 2, 13-16].

Previously, we had demonstrated that it was possible to biosynthesize different PHB copolymers by the high-performance PHAs strain-producer *Azotobacter chroococcum *7B using a variety of methodological approaches and had conducted a comprehensive study of the physico-chemical and biological properties of the resulting polymers. This strain is characterized by an ease of culturing and biotechnological process (it requires only the most basic equipment, does not require highly specific culture media, gas feeding, high-precision control of specific parameters, etc.), high productivity (high biomass yield, polymer and dry biomass content in cells up to 80% and above), and high molecular weight of the synthesized polymer (more than 1.5 × 10^6^ Da). These characteristics are extremely important for the biotechnological production of polymers for biomedical applications, since they require technically simple and deep purification, in addition to an assured efficient production [15, 21]. However, these strain-producers have certain limitations in the synthesis of PHB copolymers containing monomers of 3-hydroxycarboxylic acids with a chain length of more than five carbon atoms [22, 23]. The biosynthesis of a new PHB copolymer, poly(3-hydroxybutyrate-co- 3-hydroxy-4-methylvalerate), has been demonstrated using such bacterial producers as *Ralstonia eutropha*,* Burkholderia *sp., *Chromobacterium *sp., which can biosynthesize PHAs with short- and long-chain monomers of carboxylic acids [24-27]. However, the chemical structure of the copolymer (its monomer, 3-hydroxy- 4-methylvalerate, has a Y-shaped R-group) makes it particularly interesting for the study of its biosynthesis by such bacterial strain-producers as *Azotobacter *sp. due to these restrictions.

The possibility of biosynthesizing new PHB copolymers through such bacterial strain-producers as* Azotobacter *sp. is of great scientific and practical interest. We examined the possibility of biosynthesizing a new PHB copolymer, poly(3-hydroxybutyrate-co- 3-hydroxy-4-methylvalerate), by the highly efficient PHA strain-producer *A. chroococcum *7B, determined its physico-chemical properties, as well as its *in vitro* biocompatibility.

**Fig. 1:**
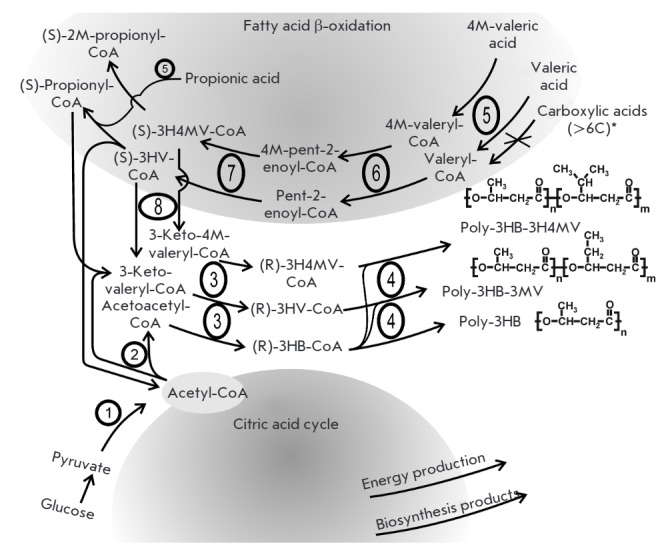
Scheme of biosynthesis of PHB and its copolymers by *A. chroococcum *7B. 1 – pyruvate dehydrogenase complex; 2 – β-ketothiolase; 3 – NADPH-dependent acetoacetyl-CoA reductase; 4 – short chain carboxylic acids PHA-polymerase; 5 – acyl-CoA synthase; 6 – acyl-CoA dehydrogenase; 7 – enoyl-CoA hydratase; 8 – NADH-dependent acetoacetyl-CoA reductase. Abbreviations: 4M – 4-methyl-; 2M – 2-methyl-; 3HB – 3-hydroxybutyrate-; 3HV – 3-hydroxyvalerate; 3H4MV – 3-hydroxy-4-methylvalerate; Poly-3HB-3H4MV – poly(3-hydroxybutyrate-co-3-hydroxy- 4-methylvalerate); Poly-3HB-3HV – poly(3-hydroxybutyrate-co-3-hydroxyvalerate); poly-3HB – poly(3-hydroxybutyrate).

## MATERIAL AND METHODS

**Reagents**

Sodium salt of valeric acid or sodium valerate (VA), sodium salt of 4-methylvaleric acid or sodium methylvalerate (4MVA), sodium salt of hexanoic acid or sodium hexanoate (HxA); components of the culture medium: K_2_HPO_4_·3H_2_O, MgSO_4_·7H_2_O, NaCl, Na_2_MoO_4_·2H_2_O, CaCO_3_, FeSO_4_·7H_2_O, sodium citrate, CaCl_2_, KH_2_PO_4_, sucrose, agar, phosphate-buffered saline (PBS). All reagents were purchased from Sigma Aldrich (Germany) and used “as purchased.”

**Biosynthesis of polymers**

Highly efficient PHB strain-producer *A. chroococcum* 7B, non-symbiotic nitrogen-fixing bacteria capable of overproducing the polymer (up to 80% of the cells’ dry weight) was used for polymer biosynthesis [28-31]. The strain was isolated from the rhizosphere of wheat (sod-podzolic soil) and maintained in Ashby’s medium containing 0.2 g/l K_2_HPO_4_·3H_2_O, 0.2 g/l MgSO_4_·7H_2_O, 0.2 g/l NaCl, 0.006 g/l Na_2_MoO_4_·2H_2_O, 5.0 g/l CaCO_3_, 20 g/l of sucrose and 20 g/l agar. All experiments were carried out under laboratory conditions. To achieve high productivity, the culture of *Azotobacter *cells was grown in shake flasks on a microbiological Innova 43 shaker (New Brunswick Scientific, USA) with constant stirring and at 30 ° C in Burk medium under conditions of excess car bon source in a medium containing 0.4 g/l MgSO_4_·7H_2_O, 0.01 g/l FeSO_4_·7H_2_O, 0.006 g/l Na_2_MoO_4_·2H_2_O, 0.5 g/l sodium citrate, 0.1 g/l CaCl_2_, 1.05 g/l K_2_HPO_4_·3H_2_O, 0.2 g/l KH_2_PO_4_ and 17 g/l (50 mM) sucrose as the main carbon source. The volume of the medium in the flask was 100 ml, which at high productivity of the *A. chroococcum* 7B strain with sampling at the end of the experiment allows one to analyze the biosynthetic processes and have a sufficient number of samples for statistical processing (each experiment was performed in eight replicates). The salts of carboxylic acids (propionic, valeric, 4-methylvaleric, hexanic) were added to the culture medium as additional carbon sources for the biosynthesis of the PHB copolymers. VA in a concentration of 5 and 20 mM was added to the culture medium immediately and after 12 hours of culturing as a monomer precursor of 3-hydroxyvaleriate within the PHA composition. These concentrations and time points were selected to produce PHBV copolymers with different contents of 3-hydroxyvaleriate in the copolymer chain [28, 29]. 4MVA and HxA were added to the culture medium as potential monomer precursors of 3-hydroxy-4-methylvalerate and 3-hydroxyhexanoate in the composition of the synthesized PHAs at a concentration of 20 mM at 0 hour and concentration of 5, 10, 20 and 35 mM after 12 hours of culturing of the strain-producer. These concentrations of the carboxylic acid were selected by analogy with the other carboxylic acids used for the biosynthesis of new PHB copolymers and according to [24-27, 29]. The strain-producer was cultured for 72 hours. The optical density of the culture medium was monitored by nephelometry. The growth and accumulation of the polymer was also monitored by light microscopy using a Biomed-1 microscope (“Biomed”, Russia) with a digital camera. The parameters of the copolymers biosynthesis: the biomass yield (g/l medium) and total polymer content in the cells (% by weight of dry cell weight) (Table 1) were measured according to the previously developed techniques. The process of isolation and purification of the polymer from strain-producer biomass includes chloroform extraction, filtration, precipitation with isopropyl alcohol, purification by multiple cycles of dissolution-precipitation, and drying [28-31].

**Table 1:** The biosynthesis of PHB copolymers by A. chroococcum 7B on a sucrose-containing culture medium supplemented with salts of carboxylic acids

Substrate	Time of addition of salts of the carboxylic acid to the culture medium, h	Biomass yield, g/l of the medium	PHA content in biomass, % of dry cells’ weight	Molecular weight of PHA, kDa	Content of 3HB/3H4MB in the copolymer, mol. %
Sucrose, 50 mM	-	5.8 ± 0.6	83.4 ± 3.1	1710	0
S + 20 mM PA	12	2.2 ± 0.7*	63.3 ± 3.3*	890	2.9
S + 5 mM VA	12	4.4 ± 0.9*	76.2 ± 3.0*	1290	2.5
S + 20 mM VA	0	3.1 ± 1.3*	67.4 ± 4.6*	1020	7.8
S + 20 mM VA	12	3.5 ± 0.8*	70.5 ± 3.2*	1270	21.3
S + 20 mM 4MVA	0	2.6 ± 1.2*	71.2 ± 4.8*	620	0.04
S + 5 mM 4MVA	12	3.7 ± 0.8*	79.3 ± 3.2*	1390	0.14
S + 10 mM 4MVA	12	3.6 ± 0.9*	78.8 ± 3.4*	1340	0.23
S + 20 mM 4MVA#	12	3.4 ± 0.9*	76.7 ± 3.3*	1300	0.60
S + 35 mM 4MVA	12	2.7 ± 0.8*	71.4 ± 3.5*	1130	0.32
S + 20 mM HxA	12	2.7 ± 0.7*	64.3 ± 3.7*	1020	0

^*^P < 0.05 compared="" with="" the="" “Sucrose” (S=““) group=““, n = “8.“

^#^Experimental data obtained in conditions of PHB4MV copolymer biosynthesis for the given line are shown in Fig. 2, Fig. 4 and in Table 2.

**Study of the chemical composition of the polymer by nuclear magnetic resonance spectroscopy (NMR)**

**Fig. 2:**
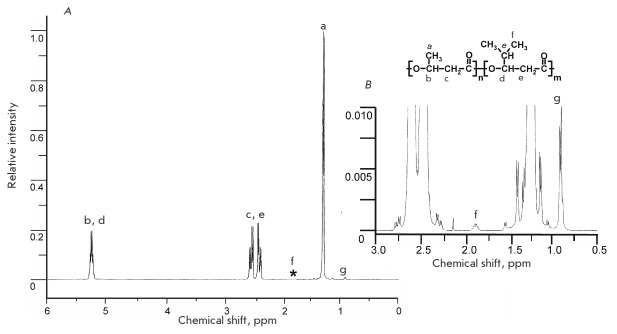
^1^H 500 MHz NMR spectrum of PHB4MV copolymer. A – PHB polymer chain: a – CH_3_ (*s*), b – CH (*b*), c – CH_2_ (*b*), poly(3-hydroxy-4-methylvalerate) polymer chain: d – CH_2_ (*s*), e – CH_3_ (*s*), f – CH (*b*), g – CH_2_ (*b*), 1 – side groups, 2 – polymer backbone; *an enlarged section of the graph is shown in the inset (*B*)

1H NMR spectra of 1% (w/v) polymer solutions in deuterated chloroform were recorded on a 300 MHz spectrometer MSL-300 (Bruker, Germany) using the following experimental parameters: temperature 313 K, relaxation delay of 2.5 s, width of the spectral window of 4,000 Hz, and a 500 MHz spectrometer Bruker Avance III with a three-channel TCI Prodigy cryodetector (Bruker, Germany) with the following experimental parameters: temperature of 310 K, relaxation delay of 3.3 s, and width of the spectral window of 10,000 Hz. The chemical shifts (in ppm) were set based on the residual CDCl3 proton signal (7.24 ppm by TMC). The percentage of 3-hydroxyvaleriate content (3HV) in the PHBV copolymer was calculated by the ratio of integrated intensities of the signal of the methyl group of the hydroxyvalerate residue (0.89 ppm) and the sum of signals of the methyl group of the hydroxyvalerate residue (0.89 ppm) and methyl group of the hydroxybutyrate residue (1.27 ppm) [29, 31]. The percentage of 3-hydroxy-4-methylvalerate (3H4MV) content in the PHB4MV copolymer was calculated by the ratio of integrated intensities of the sum of signals of the 4-methyl group (g) (0.90 ppm) and -CH group (f) (1.91 ppm) and the sum of integrated intensities of signals of the 4-methyl group and -CH-group of the 3-hydroxy- 4-methylvalerate residue and methyl group of the 3-hydroxybutyrate residue (1.27 ppm) (*Fig. 2*).

**Determination of the molecular weight of the polymers**

The molecular weights (M_w_) of the polymers were determined by gel filtration chromatography (GPC). The data obtained by GPC were correlated with viscometric data [28-31].

**Preparation of experimental samples of polymer films**

Experimental samples of polymer films with a thickness of 40 μm and diameter of 30 mm were obtained in order to study the physico-chemical properties and *in vitro* growth of cells on polymeric films. The polymers synthesized by bacteria, PHB, PHBV1 (2.5 mol% of 3HV), PHBV2 (7.8 mol% of 3HV) and PHB4MV, whose characteristics are given in Table 2, were used to produce the samples. The polymer films were prepared from a 2% (w/v) solution of the corresponding polymers in chloroform by evaporation of the solvent on a glass substrate. The weight of the films was measured using AL-64 scales (max = 60 g, d = 0.1 mg, Acculab, USA) and was 61 ± 8 mg. The film’s thickness as measured by magnetic thickness gauge was 38 ± 6 mm. Prior to working with the cell cultures, the films were sterilized by autoclaving; they were pre-incubated in distilled water at 37 °C in an incubator (EU 1/80 SPU, Russia) for 2 hours [30, 31].

**Table 2:** Physicochemical properties of the PHB copolymers obtained in A. chroococcum 7B cells.

Polymer	Chemical composition			Therophysical properties			Hydrophility
	3HV content, mol. %	Molecular mass, kDa	M_w_/M_n_	Melting point (zero and peak) (T_m_^0^/ T_m_^peak^, °C)	Crystallization point (peak) (T_c_^peak^, °C)	Crystallinity (X_c_), %	Contact angle, °
PHB	0	1710	1.7	166.8/176.9	62.2	86.6*/74.7**	70.1 ± 2.6
PHBV1	2.5	1290	1.9	166.0/174.8	60.3	56.4/52.8	70.7 ± 2.2
PHBV2	7.8	1020	1.8	161.2/169.0	66.3	47.5/45.2	76.4 ± 2.3*
PHB4MV	0.6	1300	2.0	169.9/177.3	75.1	58.0/49.6	75.1 ± 1.1*

^*^Calculated for the first heating cycle.

^**^Calculated for the second heating cycle.

Note. All columns except the last one contain mean data calculated for three measurements; in the last column “contact angle” – * p < 0.05 when="" compared="" to="" PHB="" group="", n = "10."

**Differential Scanning Calorimetry**

The thermophysical characteristics of the polymer films (melting point and crystallization point, melting heat and crystallization heat) were measured by differential scanning calorimetry according to [32, 33]. The temperatures of start and maximum of the melting peak or crystallization were designated as T_m_^0^, T_m_^peak^ and T_c_^peak^ , respectively. PHA crystallinity (*Xc*) was calculated according to [33]:

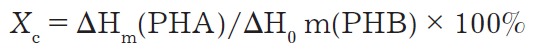



where ΔH_0_ m(PHB) is the theoretical value of the thermodynamic melting enthalpy, which for 100% crystalline PHB could be 146.6 J/g [34], and ΔH_m_(PHA) is the experimental melting enthalpy of the corresponding sample of PHA. Calculations of the degree of crystallinity and melting points of the samples were generated for the data obtained in the second polymers heating cycle; the crystallization temperature is based on the data obtained in the first cooling cycle. The data are presented as mean values of three measurements.

**Contact angle measurement**

The hydrophilicity of the polymer films’ surface was evaluated by measuring the contact angle formed between a water droplet and the polymer film’s surface, using a digital inclinometer, Drop Shape Analysis System DSA100 (KRUSS, GmbH, Germany), according to [30, 31].

**Study of the stromal cells growth on polymer films**

Stromal cells (BMSCs) were isolated from bone marrow femurs of 3-day-old Wistar rats according to standard procedures [35]. The animals were killed by decapitation, the femurs were removed, epiphyses were cut, and bone marrow was washed out of the diaphysis with a syringe (2 mm, 27G needle). The resulting suspension was incubated in a DMEM medium with type 1 collagenase (1075 U/ml) (“PanEco”, Russia) for 1 h at 37 °C, centrifuged (10 min, 100 rpm), and the precipitate was precipitated on the culture plastic. The growth medium was changed the next day, and the cells were further cultivated until the formation of a primary monolayer culture.

The cell viability was assessed using the XTT test, an analogue of the widely used MTT test [30, 31, 36]. This test is based on the conversion of uncolored tetrazolium salts into colored formazan compounds by the action of NADPH-dependent oxidoreductases, and it allows one to evaluate the activity of mitochondrial dehydrogenases. We used the XTT set (XTT Cell Proliferation Kit, Biological Industries, Israel).

The aim of our work was not to check the cytotoxicity, but to identify cell proliferation in matrixes: i.e., the biocompatibility of the polymeric films. The cells were maintained in a DMEM medium (Dubecco’s Modified Eagle Medium, “PanEco”, Russia) with 10% fetal calf serum (Biological Industries, Israel), 100 IU/ml penicillin and 100 μg/ml streptomycin (“PanEco”) at 37 °C in atmosphere with 5% CO_2_. The medium was changed every 3 days. The sterile samples of PHB, PHBV1, and PHB4MV films (sterilization by autoclaving) (*n *= 6) were placed into the wells of a 96-well plate, and the cell suspension was applied to the top in a concentration of 1,500 cells per sample. The second-passage cells were used, since the proliferation of the first-passage cells was not fully stable, and there were significant differences in the growth of the first-passage cells on polymeric films in repeated experiments. We determined the viability of the cells cultured on the polymer films after 1, 3, 7 days as it was important to assess the dynamics of this parameter. The growth of the cells was stable within this time interval, and the data points allow one to most comprehensively describe the dynamics of BMSCs growth on the films. The culture medium was removed from the wells after the pre-determined time, 100 μl of fresh medium was added into new clean wells, and our samples were transferred therein. This was done in order to take into account only the cells attached to the polymer substrate and to ignore the cells that could detach from the substrate and attach to the polymeric plate. 50 μl of a freshly prepared XTT solution (as described) was added to the wells. After 4 h of incubation at 37 °C with gentle rocking, the samples were removed and their optical density was measured on the Zenyth 3100 Microplate Multimode Detector (Anthos Labtec Instruments GmbH, Austria) at 450 nm against 690 nm [30, 31].

**Statistical analysis**

Statistical processing of the polymers’ biosynthetic parameters, their contact angles and *in vitro *biocompatibility in a cell culture was performed using the SPSS/ PC+ Statistics™ 12.1 (SPSS) software package. One-way ANOVA was used. The data in the tables and in the figures are presented as mean values and standard error of the mean (M ± SD) at a significance level of P < 0.05. The number of measurements (*n*) is given in the figure captions and footnotes to the tables. The mean values of the polymers’ physico-chemical properties calculated from the three measurements are presented.

## RESULTS AND DISCUSSION

**Biosynthesis of PHB copolymers using additional sources of carbon**

The results of the study of PHB copolymers biosynthesis by the strain-producer *A. chroococcum *7B in the presence of various additional carbon sources in a culture medium (salts of propionic, valeric, 4-methylvaleric, and hexanoic acids) are shown in *Table 1*. The results of the PHBV copolymer biosynthesis study confirm previously obtained data: 3-hydroxyvaleriate monomers are incorporated into the PHBV copolymer chain if valeric and propionic acids are used as additional carbon sources, whereas the presence of a longer chain hexanoic acid does not result in the synthesis of a copolymer. The molar content of 3HV in the synthesized copolymer is directly proportional to the concentration of the VA added to the culture medium. The molecular weight of the PHBV polymer was lower than that of the PHB homopolymer, which is probably due to the inhibitory effect of valerate on the polymer synthesis. If sucrose is the only carbon source in the medium, the strain produces high-molecular PHB (1710 kDa) [29, 37-39].

Various additional carbon sources are used in order to improve the parameters of polymer biosynthesis. It has been shown that additional carbon sources not only influence the molecular weight of the synthesized polymers, but also result in the synthesis of new copolymers with modified physicochemical and biomedical properties [29-31, 40-46].

Using this method, we demonstrated the possibility of biosynthesizing the PHB4MV copolymer, a novel one for the strain-producer *A. chroococcum *7B, by adding 4MVA as an additional carbon source and a precursor of the 3H4MV monomer in the copolymer chain to the culture medium. The incorporation of 3H4MV residues into the synthesized PHB4MV polymer was also confirmed by 1H NMR spectroscopy data. In the 1H NMR spectrum, the 4-methyl group (f) and -CH-group (g) of the 3H4MV monomer are represented by signals at 0.90 and 1.91 ppm, respectively (*Fig. 2*), whereas the PHB homopolymer and PHBV copolymer have no signals in this range. We assume that, similarly to PHBV, the obtained copolymer is a multiblock copolymer and its synthesis proceeds as follows: 4MVA → 4-methylvaleryl-CoA → 3-keto-4-methylvaleryl-CoA → *D*-3-hydroxy- 4-methylvaleryl-CoA → 3H4MV in the composition of PHB4MV; i.e. similarly to PHBV biosynthesis: VA → valeryl-CoA → 3 ketovaleryl-CoA → *D*-3-hydroxyvaleryl- CoA → 3HV as part of PHVB [29, 37-39] (*Fig. 1*).

The maximum incorporation of 3H4MV monomers into the synthesized PHB4MV polymer is 0.6 mol% for the case when 4MVA is added to the culture medium in a concentration of 20 mM as an additional carbon source; at other concentrations of the precursor carboxylic acid, the incorporation of monomers was much lower. Nevertheless, synthesis of this copolymer is confirmed.

*PhbC*-encoded PHB synthase is a polymerase of short-chain carboxylic acids, such as 3-hydroxybutyrate and 3-hydroxyvalerate. This polymerase is unable to utilize medium- and long-chain 3-hydroxycarboxylic acids, namely those longer than 3-hydroxyvaleric acid (5C 3-hydroxycarboxylic acid), to synthesize PHAs: i.e., this enzyme cannot incorporate 3-hydroxyhexanoic acid and 3-hydroxyheptanoic acid into the growing PHA chain [22, 23]. Nevertheless, we used HxA as an additive, because as a 4MVA isomer it can serve as a control because it is known that the presence of HxA does not lead to the synthesis of the PHB copolymer by* A. chroococcum *cells. The effect of HxA in itself on the biosynthesis process had to be controlled, though. Our data confirm the restriction on the length of the monomers used by PHB-synthase, which appears to be associated with the strict specificity of this enzyme with respect to the substrates used for polymer synthesis. Incorporation of 3-hydroxy-4-methylvalerate residues only confirms this restriction, because in spite of the fact that 3-hydroxy-4-methylvalerate is a residue of 6C 3-hydroxycarboxylic acid, its side chain is branched and, therefore, the length of the side chain is not increased. However, a linear molecule, 3-hydroxyhexanoic acid (6C linear 3-hydroxycarboxylic acid), cannot be incorporated into the growing polyester chain by the enzyme, for the same reasons.

Interestingly, the addition of VA and 4MVA to the culture medium causes a slight decrease in the molecular weight of the synthesized polymer that can be explained by an inhibitory effect of carboxylic acids on PHA biosynthesis (*Table 1*). However, the addition of 4MVA to the culture medium immediately, rather than after 12 hours, results not only in a considerable decrease in the molecular weight of the polymer, but also the PHB4MV copolymer is hardly produced at all. A similar effect was observed in the case of initial addition of VA to the culture medium, which resulted in the synthesis of a PHBV copolymer with a much lower content of 3HV monomers. Reduction of the molecular weight is observed even in the case of addition of HxA to the culture medium, although the corresponding copolymer is not synthesized. This may also be due to the inhibitory action of carboxylic acids on PHB-synthase, which leads to a decrease in the incorporation of the molecular precursor into a growing copolymer chain in the early stages of polymer biosynthesis, even though in theory it must, in contrast, lead to the synthesis of copolymers with a higher content of 3HV and 3H4MV.

The effect of carboxylic acids on polymers biosynthesis is confirmed by the results of the study of *A. chroococcum *7B culture growth. The results obtained indicate that the addition of carboxylic acids to the medium results in a marked inhibition of cell growth, reduced polymer content, and, consequently, polymer production, and the degree of the inhibitory effect on cell growth depends on the nature of the chemical additive [29]. For example, despite the fact that the use of HxA as an additional carbon source does not lead to a copolymer synthesis, HxA significantly inhibits cell growth and the production of the polymer (*Table 1Table 1*).

Despite a slight decrease in PHB4MV biosynthesis parameters, the high productivity (biomass yield, 3.4 g/l; copolymer content, 76.7%) of the strain-producer and high molecular weight of the copolymer (1.3 × 106) should be noted. Biosynthesis of PHB4MV has previously been demonstrated using different producers: *R. eutropha*, *Burkholderia *sp, *Chromobacterium* sp. However, the polymer content in the cells of the producer-strains rarely exceeded 50% and the biotechnological process required highly specific technical conditions that may significantly restrict the use of these techniques for the production of new polymers for biomedical applications. The biocompatibility of the synthesized copolymers was not tested, probably due to the challenges posed by the developed techniques [24-27]. Therefore, it appears particularly important to use a highly productive and hardy strains-producer such as *A. chroococcum *7B to obtain novel copolymers.

**Fig. 3:**
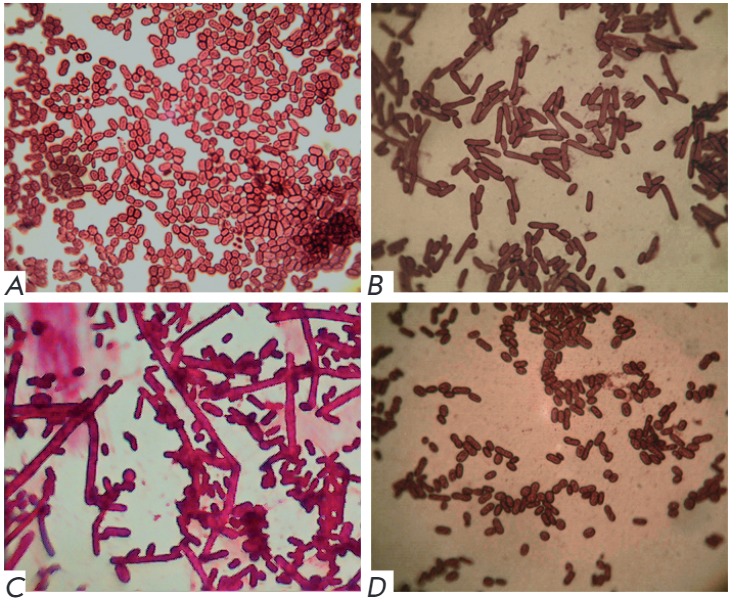
The effect of adding carboxylic acids to the culture medium on the morphology of strain-producer *A. chroococcum* cells (light microscopy, ×900). A – S + 5 mM VA (added after 12 hours), after 72 hours of culturing; B – S + 20 mM VA (added t 0 h) after 72 h of culturing; C – S + 20 mM HxA (added after 12 hours) after 72 hours of culturing; D – S + 20 mM 4MVA (added after 12 hours), after 72 hours of culturing

The addition of carboxylic acids to the culture medium also causes changes in the morphology of bacterial cells (*Fig. 3*). *A. chroococcum* is characterized by a high tendency toward cell pleomorphism, and this effect can be attributed to it. For example, if valeric acid was added in low concentrations (5 mM) the morphology would remain almost unchanged, but the addition of VA in relatively high concentrations (20 mM) resulted in a marked change in cell morphology: coccoid cells were transformed into bacillar forms (*Fig. 3B*). The addition of 20 mM HxA resulted in the appearance of filamentous cells, even though coccoid and bacillary forms were also present (*Fig. 3B*). This effect of carboxylic acids on the morphology of bacterial cells is similar to the well-known effect of various stress-inducing agents (acids, alkalis, peptone) on a cell’s shape [47, 48].

**Study of the physico-chemical properties of the polymers**

The study of the physico-chemical properties of the polymers synthesized by strains of *A. chroococcum *7B revealed a significant difference between the thermo- physical and hydrophilic properties of PHB copolymers, PHBV1 (2.5 mol% 3HV), PHBV2 (7.8 mol% 3HV) and PHB4MV, as well as PHB homopolymer, despite the low molar content of 3H4MV and 3HV in the PHBV1 and PHB4MV copolymers, respectively (*Table 2*).

**Fig. 4:**
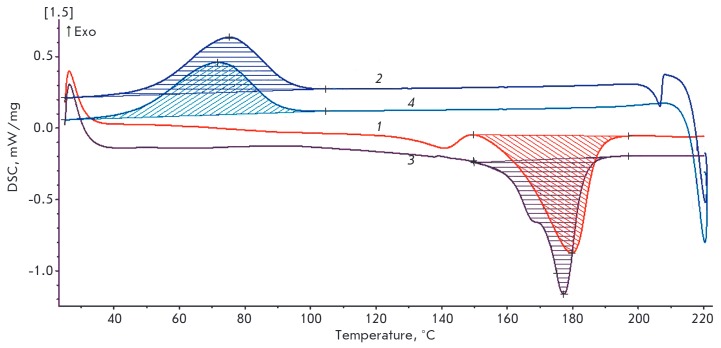
DSC thermograms of PHB4MV obtained by biosynthesis by *A. chroococcum *7B: 1 – curve of the first heating cycle; 2 – curve of the first cooling cycle; 3 – curve of the second heating cycle; 4 – curve of the second cooling cycle; areas of the melting and crystallization peaks are shaded, respectively

*Fig. 4*shows the DSC thermograms of the PHBV and PHB4MV copolymers compared to PHB. The thermogram of polymers melting contains the expressed melting peaks of semi-crystalline polymers and their crystallization peaks. The melting peaks of the PBHV and PHB4MV copolymers compared to the PHB homopolymer are characterized by:

– a slight change in the melting peak, indicating the absence of a significant change in the melting point of the copolymers;

– a shift of the PHB4MV crystallization peak to higher temperatures, indicating an increase in the crystallization temperature of the copolymer; and

– a decrease in the area of the melting peak, indicating a decrease in the melting enthalpy and accordingly crystallinity of the copolymers.

Calculation of the thermo-physical parameters obtained from the analysis of DSC thermograms data is shown in *Table 2*. As can be seen both the PHBV and PHB4MV copolymers have a much lower degree of crystallinity than PHB (21.9 and 25.1%, respectively), and the new PHB4MV copolymer has an even larger drop in the degree of crystallinity than the PHBV1 copolymer, even though the molar content of 3H4MV in PHB4MV is only 0.6 compared to 2.5% of 3HV in PHBV. However, PHB4MV has a degree of crystallinity comparable to that of the PHBV2 copolymer, in which the molar content 3HV is 7.8%. Partially, this drop in the crystallinity of the copolymers may be due to a lower molecular weight (by more than 300 kDa in comparison with PHB). The crystallinity indicators (calculated from the first and second cycles of heating of the polymer samples, see *Table 2*) are in agreement with the published data [49]. A decrease in the molecular weight of the polymers has been shown to lead to a quite significant drop in the degree of crystallinity (10% or more if Mw is reduced two-fold) [49]. However, the main role in the drop in the degree of crystallinity is played by the monomers (3HV and 3H4MV) in the copolymers with a side group longer than that of 3HB. This confirms the data that introduction of 3HV monomers into a PHB polymer chain results in a copolymer with altered physico-chemical properties: a lower melting point, lower crystallinity, higher plasticity, and lower durability and higher biodegradation rate [22, 32], and that the crystallinity of the PHBV copolymer decreases significantly with the increase in the molar content of the 3HV monomers in its chain [32]. However, in the case of PHB4MV we observe a much more pronounced effect: in its physico-chemical properties the PHB4MV copolymer with only a 0.6% molar content of 3H4MV resembles a PHVB copolymer with a molar content of 2.5 to 7.8%. Something similar is observed in the analysis of the polymers’ hydrophilicity. While the contact angles (as an indicator of the hydrophilicity of polymer surfaces) of the PHB homopolymer and PHBV1 copolymer do not differ, the value for the PHBV2 and PHB4MV copolymers was considerably higher and the contact angle of PHB4MV was only slightly lower than that of PHBV2. We have previously shown that the contact angle of the PHBV copolymer increases with an increase in the molar content of 3HV monomers, and that the hydrophilicity of a polymer film is reduced due to an increased concentration of hydrophobic groups on its surface [50]. Thus, based on the data of the analysis of the hydrophilic properties of the polymers, the PHB4MV copolymer containing only 0.6% of 3H4MV corresponds to a PHBV copolymer with a molar content of 2.5 to 7.8%. This may be due to a much more pronounced destabilizing effect of the branched side group (3H4MV residue) on the crystal structure of the polymer compared with the effect of the linear 3HV group in the PHVB copolymer (*Fig. 1*), which explains such a disproportionately large contribution of the low content of 3H4MV to the change in the physico-chemical properties of the polymer.

**Investigation of the growth of stromal cells on the polymer films**

**Fig. 5:**
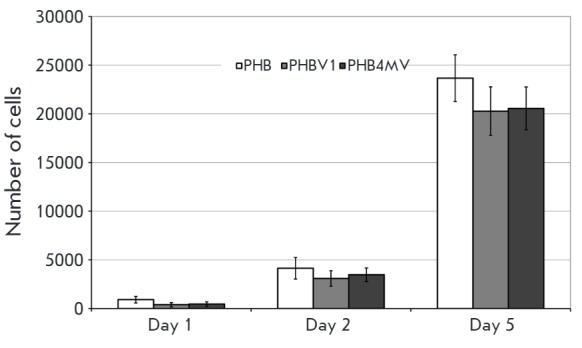
Changes in the number of viable bone marrow stromal cells of rats cultured on PHB, PHBV1 and PHB4MV polymer films according to the XTT test. * P < 0.05 when compared to PHB group, *n *= 6

Studies of the *in vitro *biocompatibility of polymers produced by biosynthesis in *A. chroococcum *7B cells using a culture of the stromal cells isolated from bone marrow revealed a significant increase in the number of viable BMSCs on the films of three polymers, PHB, PHBV1 (2.5%mol 3HV), and PHB4MV, over 5 days (Fig. 5). No statistical differences were observed in the cell proliferation on the films of the different polymers. Therefore, the new PHB4MV copolymer can be used for biomedical research and development, along with its analogues – PHB and PHBV – particularly for the manufacture of matrices used in bone tissue engineering [51, 52].

## CONCLUSIONS

We have shown that the addition of 4-methylvaleric acid to a culture medium of the strain-producer* A. chroococcum *7B leads to the incorporation of the 6C-hydroxycarboxylic acid monomer, 3-hydroxy- 4-methylvalerate, into the polymer chain of PHB, and the synthesis of poly(3-hydroxybutyrate- co-3-hydroxy-4-methylvalerate). Despite the low molar content of 3H4MV in the obtained copolymer, the physico-chemical properties of PHB4MV containing only 0.6% of 3H4MV are comparable to those of a PHBV copolymer containing 2.5 to 7.8% of 3HV. The growth of the BMSCs as determined by the XTT test on the PHB4MV copolymer *in vitro *did not differ significantly from their growth on PHB and PHBV, and, therefore, it can be used in biomedical research and development.
